# Correction to: Genetic Diversity in Chimpanzee Transcriptomics Does Not Represent Wild Populations

**DOI:** 10.1093/gbe/evaf168

**Published:** 2025-09-04

This is a correction to: Navya Shukla, Bobbie Shaban, Irene Gallego Romero, Genetic Diversity in Chimpanzee Transcriptomics Does Not Represent Wild Populations, *Genome Biology and Evolution*, Volume 13, Issue 11, November 2021, evab247, https://doi.org/10.1093/gbe/evab247

In the published paper, part of the legend for Figure 6 appears within the body of the article text, as the fourth paragraph, under subheading **Identifying Unique and Unrelated Individuals in Transcriptomic Data Sets**. The full text for Figure 6 should read:

“**Fig. 6.** Genetic diversity and sample swaps amongst iPSC and iPSC-associated samples.

Genetic diversity, tissue type, and relationships amongst the 15/14 chimpanzee iPSC lines in our data set. Ancestry estimates are as in figures 2 and 5. Samples that appear to be mislabeled are indicated by the red box next to individual sample IDs, multiple samples predicted to be from the same genetically unique individual are marked in the final ID column.”

The emendation is outlined in this notice only to preserve the version of record.

