# Supplementary material for: Genetic Diversity in Chimpanzee Transcriptomics Does Not Represent Wild Populations
Source: Genome Biol Evol. 2021 Nov 12;13(11):evab247. doi: 10.1093/gbe/evab247 (PMC8633730; doi:10.1093/gbe/evab247)
Supplement: evab247_Supplementary_Data [file evab247_supplementary_data.zip › List of Supplementary Table and Figures_210832.docx]

# Supplementary Table and Figures

Supplementary information consists of 6 tables and 7 figures.

- **Supplementary Table 1: List of studies**. All studies considered in the sampling process and relevant meta-information.
- **Supplementary Table 2: List of samples**. All chimpanzee-identified samples from 40 studies, including un-analysed replicates. A “Sample_” name was used for every sample during processing.
- **Supplementary Table 3: Mislabelled samples not of chimpanzee origin**. Samples determined to be mislabelled from the results of clustering analysis.
- **Supplementary Table 4: Unexpected relative pairs from Somalier analysis.**
- **Supplementary Table 5: Known and inferred relative pairs from IBD analyses**.
- **Supplementary Table 6: SNPs preserved at missingness thresholds of 1%, 2% and 5%, for both RNA-seq data merged to reference SNPs and unmerged RNA-seq data:**
- **Supplementary Figure 1. 486 chimpanzee-identified samples in public RNA-seq data.** UPGMA dendogram was generated from all genotyped samples. 54,706 SNPs were genotyped at ≤ 5% missingness and used to calculate pairwise IBS distances between samples. Non-chimpanzee outlier samples have been indicated, forming a distinct clade from the rest of the dataset.
- **Supplementary Figure 2: Principal component analysis of 527 chimpanzee samples with genotype data at different missingness thresholds.** Projection of 468 chimpanzee RNA-seq samples of unknown ancestry onto the PCA of 59 wild-born samples of known ancestry [(de Manuel et al. 2016)](https://www.zotero.org/google-docs/?S4gluK) using **A.** 18,679 SNPs at 10% missingness. **B.** 11,659 SNPs at 5% missingness. **C.** 5814 SNPs at 2% missingness. **D.** 3545 SNPs at 1% missingness. Colors indicate the four distinct chimpanzee subspecies and samples of unknown ancestry from public RNA-seq datasets.
- **Supplementary Figure 3: Comparison of supervised and unsupervised ADMIXTURE analyses of 468 chimpanzee RNA-seq samples of unknown ancestry. The samples** from [(de Manuel et al. 2016)](https://www.zotero.org/google-docs/?GlTVx6) are shown at the top; internal study nomenclature is given in parenthesis for the 40 RNA-seq datasets with samples of unknown ancestry; full references are available in Supplementary Table 1. Within a study, samples are ordered by SRA identifier.
- **Supplementary Figure 4: 468 chimpanzee samples in public RNA-seq data.** UPGMA dendogram of 468 samples retained after removing non-chimpanzee outliers. The first column shows the tissue from which the sample was derived. The second column distinguishes different replicate sets from different studies. The particularly extensive clade of cell line PR00818 has been labelled.
- **Supplementary Figure 5: Duplicate clusters identified from 237 chimpanzee samples.** UPGMA dendogram of 237 samples, retaining only one replicate per individual in a study. Filled boxes indicate the site from which each individual was sampled (if known). Clusters of potentially duplicate samples, identified from clustering and metadata information, have been labelled numerically, bottom to top. Samples labelled in red are failing to cluster with other duplicates and discussed in the text.
- **Supplementary Figure 6: Relationships between non-clustering sample pairs with unexpectedly high Somalier-calculated relatedness.** Ternary diagrams showing pairwise IBD estimates between a subset of 25 samples. Samples were taken from the 18 non-clustering pairs with ≤ 0.5 Somalier-calculated relatedness. All possible pairs including these 25 individuals have been plotted and labelled. Thresholds for defining different relationships between pairs are indicated by black lines on the diagram.
- **Supplementary Figure 7: Site frequency spectra in RNA-seq derived genotype data. A.** In the full data set **B.** After removing known replicate samples **C.** In the full dataset, after filtering for 5% missingness.
