# Supplementary material for: Genetic Diversity in Chimpanzee Transcriptomics Does Not Represent Wild Populations
Source: Genome Biol Evol. 2021 Nov 12;13(11):evab247. doi: 10.1093/gbe/evab247 (PMC8633730; doi:10.1093/gbe/evab247)
Supplement: evab247_Supplementary_Data [file evab247_supplementary_data.zip › Supp_fig_7_ternary.pdf]

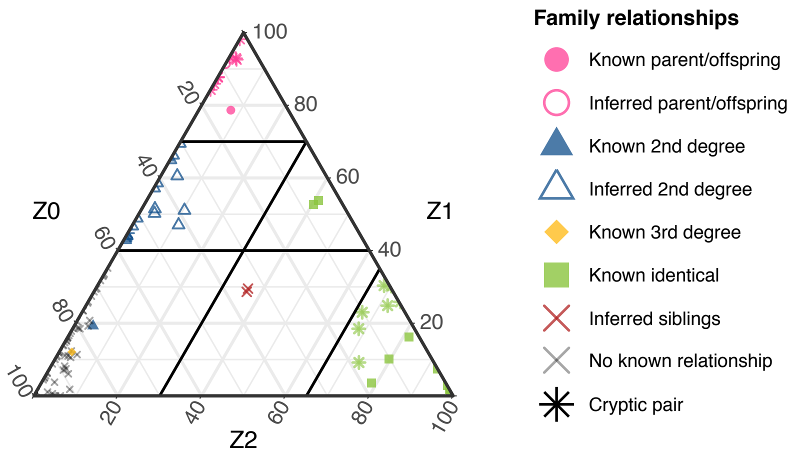
Family relationships
Known parent/offspring
Inferred parent/offspring
Known 2nd degree
Inferred 2nd degree
Known 3rd degree
Known identical
Inferred siblings
No known relationship
Cryptic pair
Z0
Z1
Z2
100
80
60
40
20
