# Supplementary figures and images for: Genetic Diversity in Chimpanzee Transcriptomics Does Not Represent Wild Populations
Source: Genome Biol Evol. 2021 Nov 12;13(11):evab247. doi: 10.1093/gbe/evab247 (PMC8633730; doi:10.1093/gbe/evab247)

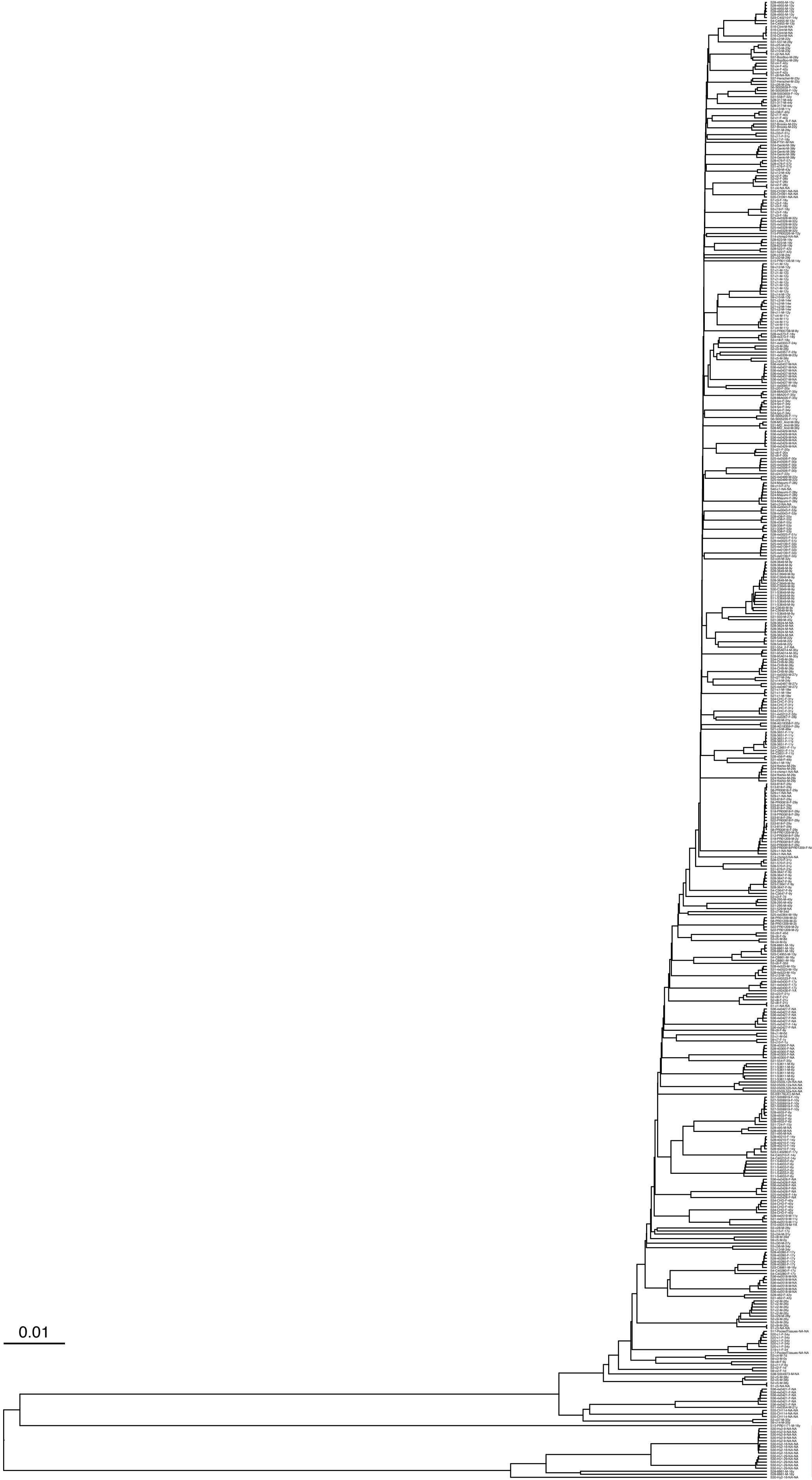

# Outliers

Supplement: evab247_Supplementary_Data [file evab247_supplementary_data.zip › Supp_fig_1_486.pdf]

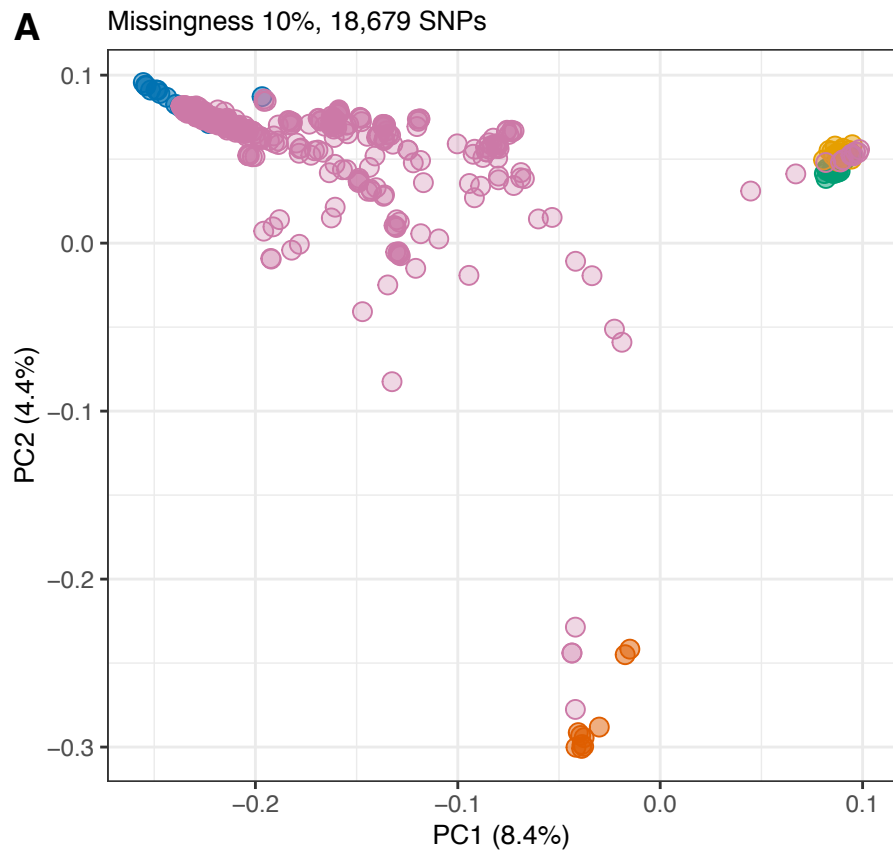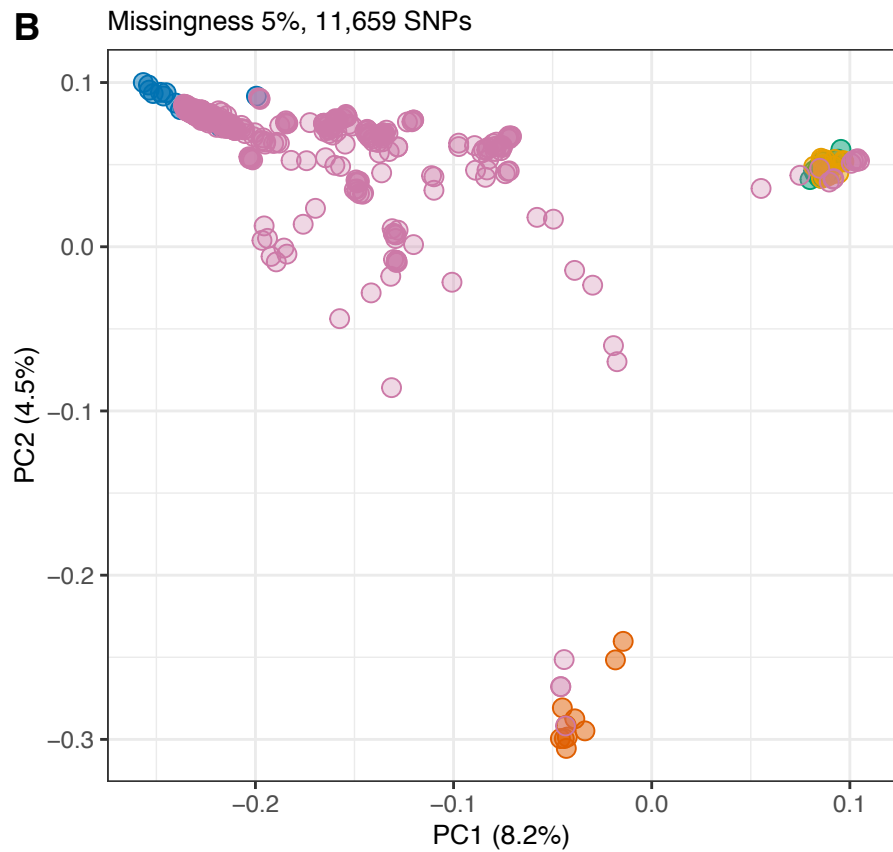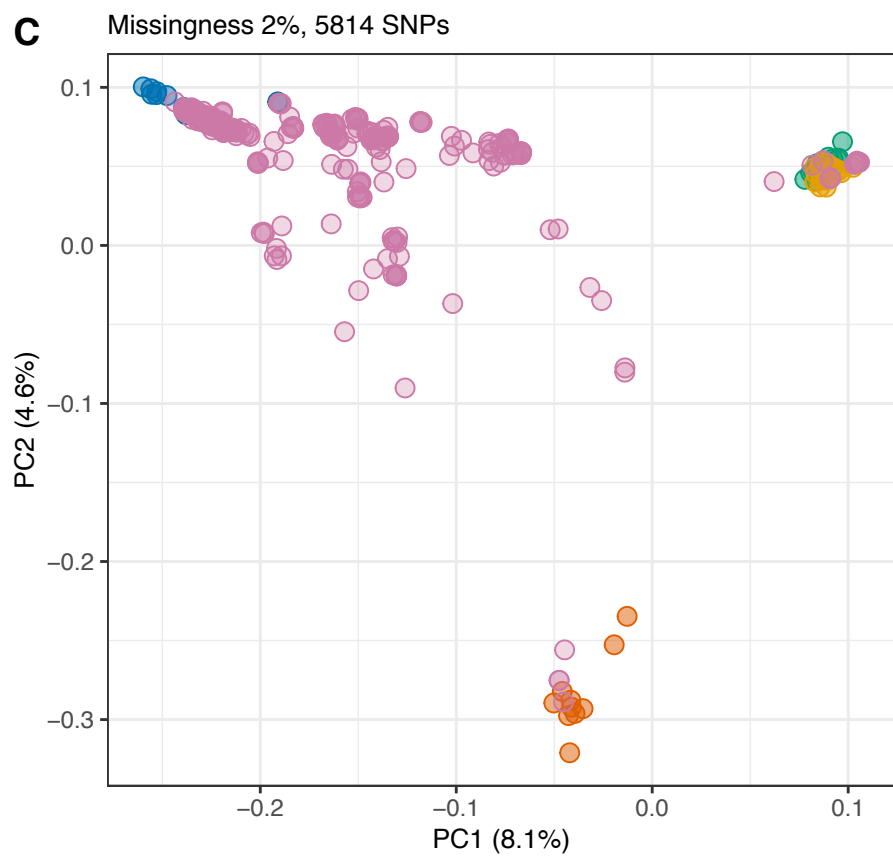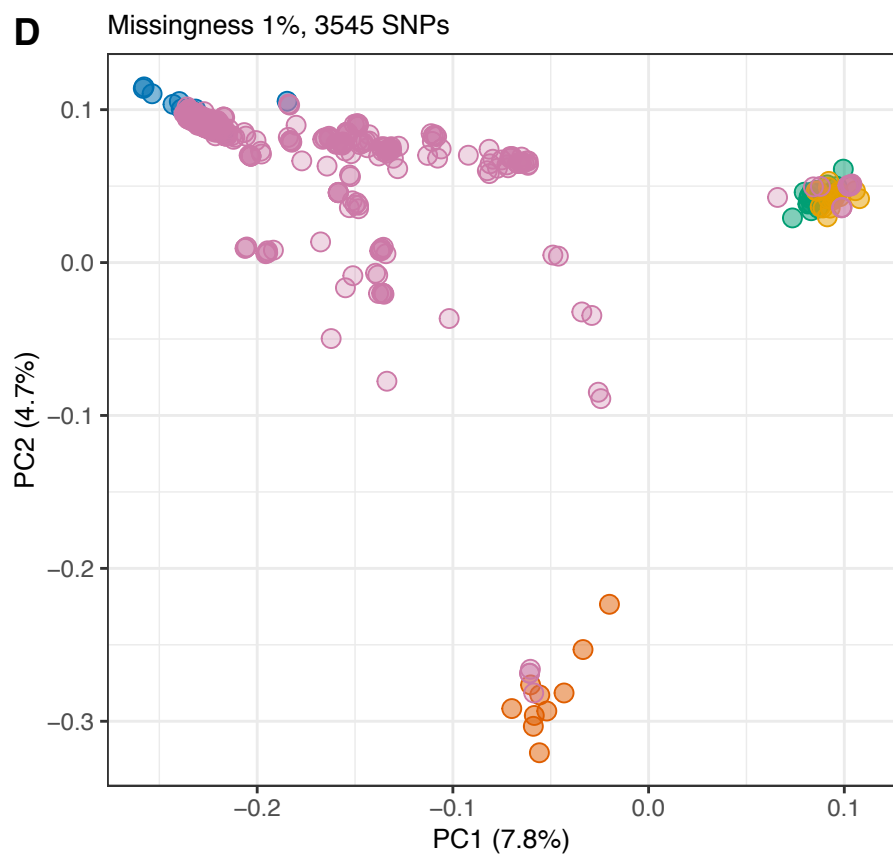

Western Nigeria-Cameroon Central Eastern RNA-seq

Supplement: evab247_Supplementary_Data [file evab247_supplementary_data.zip › Supp_fig_2_PCA.pdf]

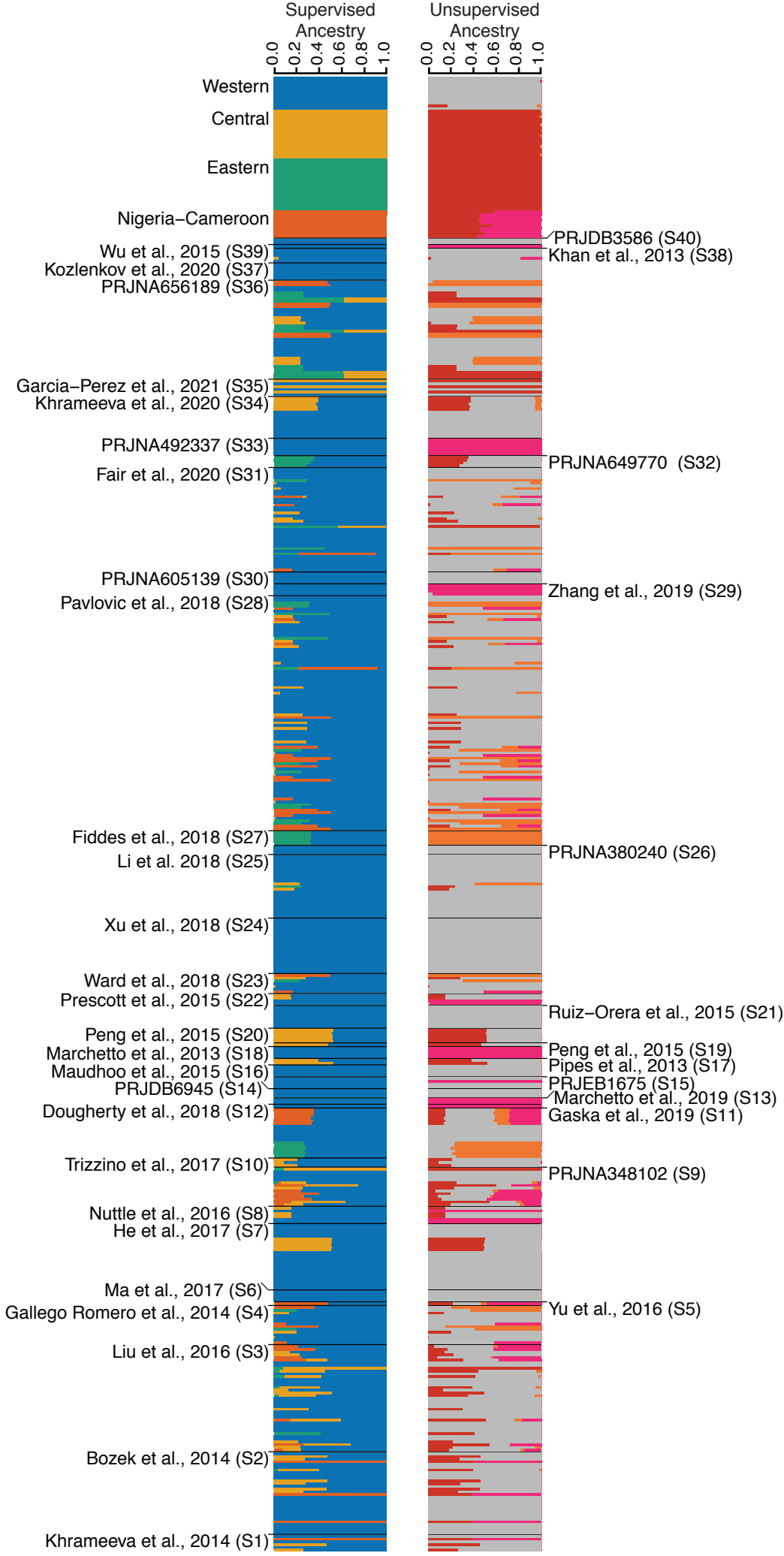

Supplement: evab247_Supplementary_Data [file evab247_supplementary_data.zip › Supp_fig_3_unsupervised_admix_altcols.pdf]

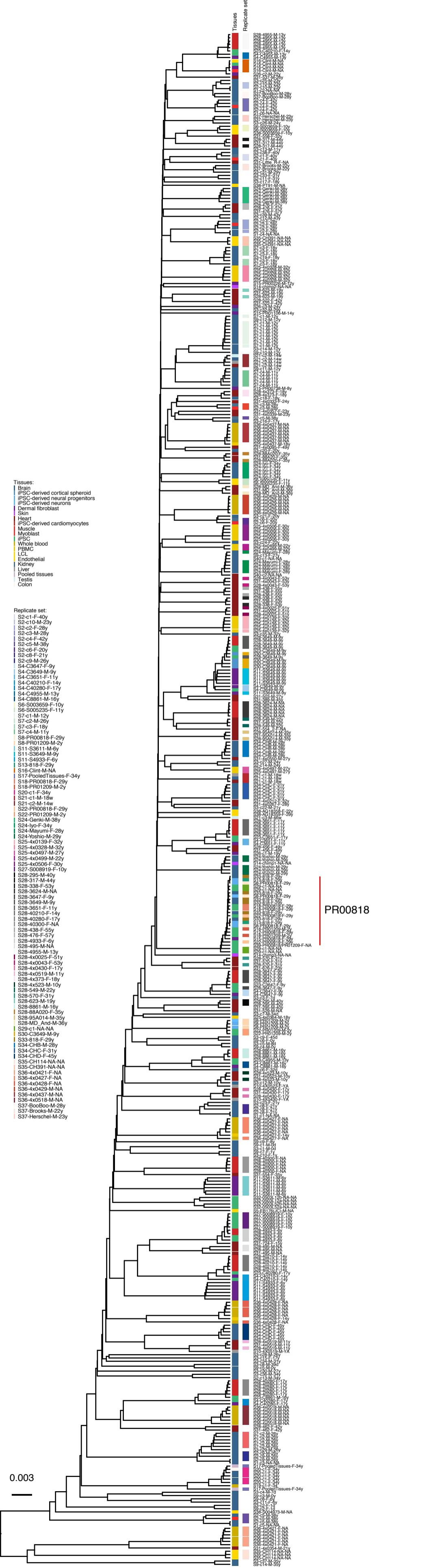

Supplement: evab247_Supplementary_Data [file evab247_supplementary_data.zip › Supp_fig_4_468.pdf]

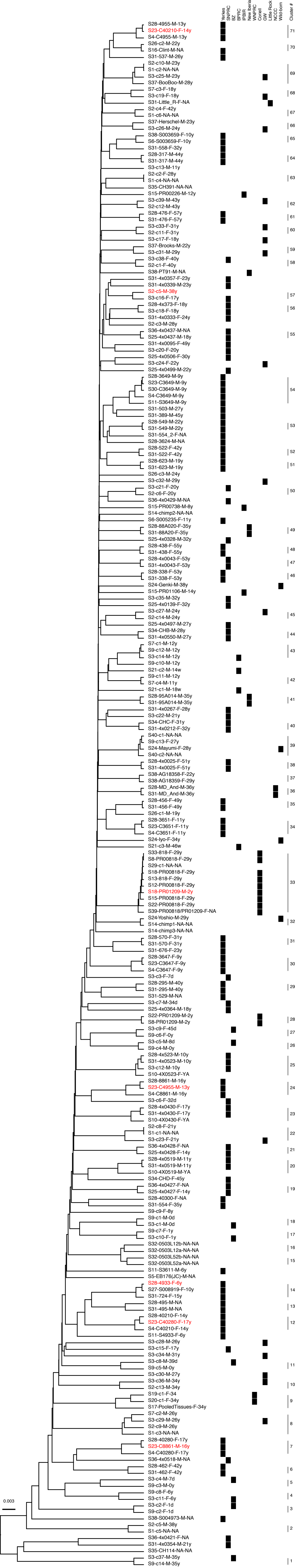

Supplement: evab247_Supplementary_Data [file evab247_supplementary_data.zip › Supp_fig_5_237.pdf]

**A**

6,943,957 SNPs, 486 individuals

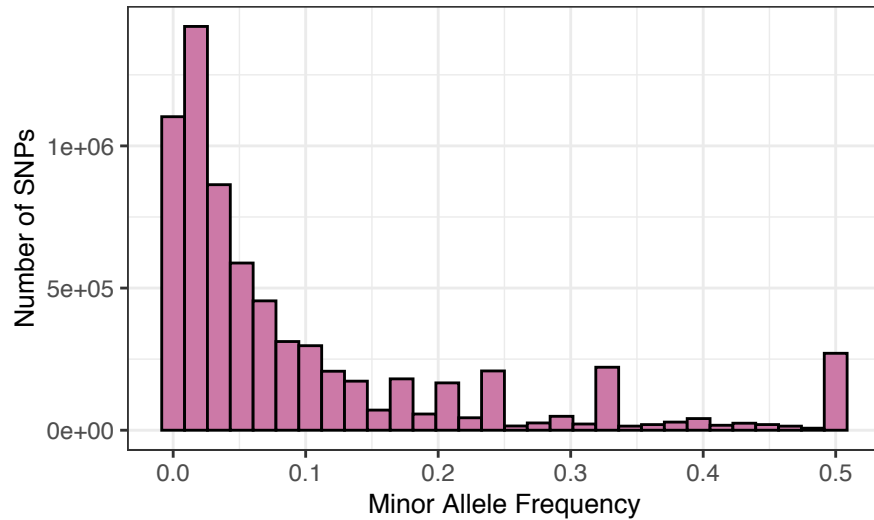**B**

6,943,957 SNPs, 237 individuals

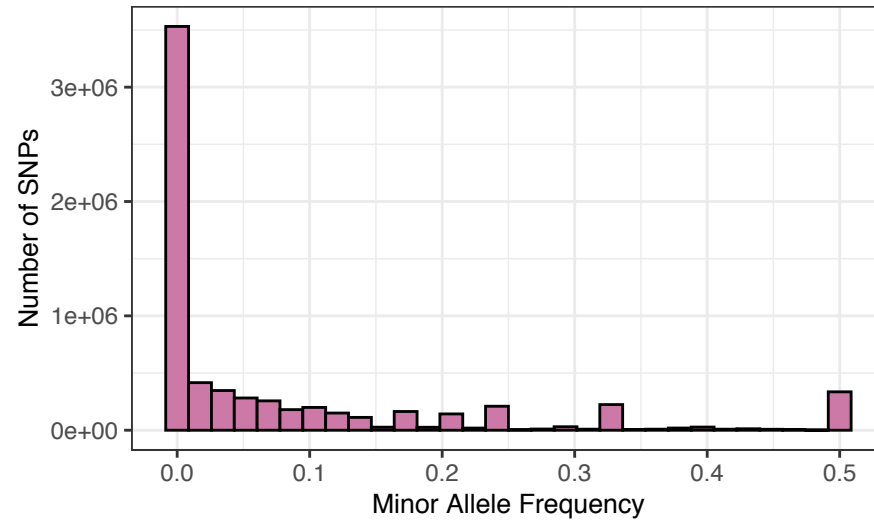**C**

Missingness 5%, 112,023 SNPs, 486 individuals

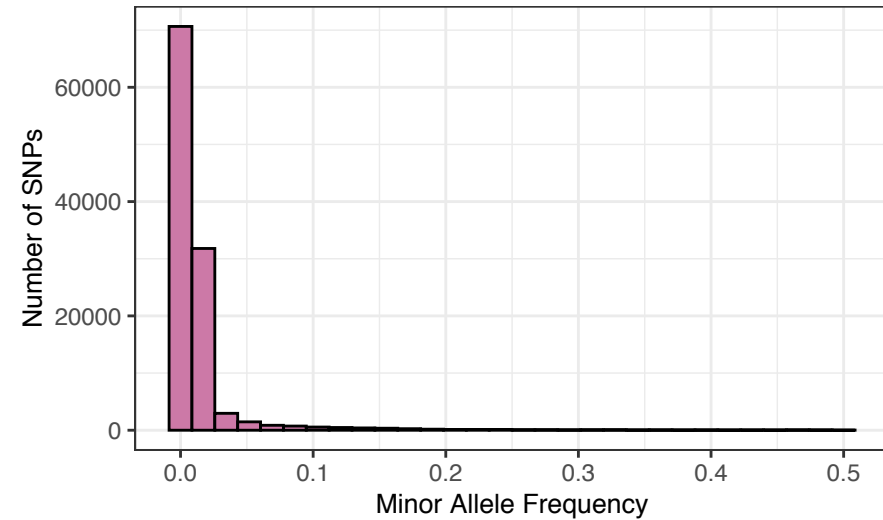

Supplement: evab247_Supplementary_Data [file evab247_supplementary_data.zip › Supp_fig_6_SFS.pdf]
